# Structural insight in the toppling mechanism of an energy-coupling factor transporter

**DOI:** 10.1038/ncomms11072

**Published:** 2016-03-30

**Authors:** Lotteke J. Y. M. Swier, Albert Guskov, Dirk J. Slotboom

**Affiliations:** 1 Groningen Biomolecular Science and Biotechnology Institute, University of Groningen, Nijenborgh 4, 9747 AG Groningen, The Netherlands; 2 Zernike Institute for Advanced Materials, University of Groningen, Nijenborgh 4, 9747 AG Groningen, The Netherlands

## Abstract

Energy-coupling factor (ECF) transporters mediate uptake of micronutrients in prokaryotes. The transporters consist of an S-component that binds the transported substrate and an ECF module (EcfAA′T) that binds and hydrolyses ATP. The mechanism of transport is poorly understood but presumably involves an unusual step in which the membrane-embedded S-component topples over to carry the substrate across the membrane. In many ECF transporters, the S-component dissociates from the ECF module after transport. Subsequently, substrate-bound S-components out-compete the empty proteins for re-binding to the ECF module in a new round of transport. Here we present crystal structures of the folate-specific transporter ECF–FolT from *Lactobacillus delbrueckii.* Interaction of the ECF module with FolT stabilizes the toppled state, and simultaneously destroys the high-affinity folate-binding site, allowing substrate release into the cytosol. We hypothesize that differences in the kinetics of toppling can explain how substrate-loaded FolT out-competes *apo*-FolT for association with the ECF module.

## Abstract (web summary)

Prokaryotes use energy-coupling factor transporters to uptake required micronutrients and an unusual toppling mechanism has been proposed for their function. Here, the authors provide structural support for this mechanism, allowing direct visualization of the toppled state.

## Graphical abstract

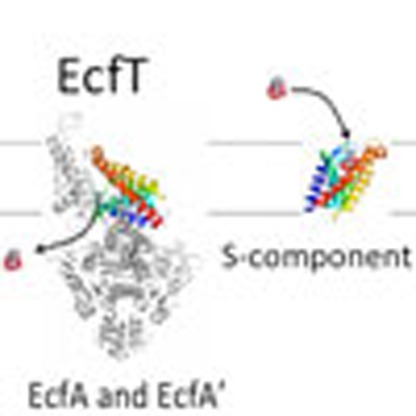

Energy-coupling factor (ECF) transporters are a recently discovered class of ATP-binding cassette (ABC) transporters^1^,^2^,^3^,^4^. The transporters are exclusively present in prokaryotes, where they catalyse the uptake of a wide variety of micronutrients, typically water-soluble vitamins and transition metals such as Ni^2+^ and Co^2+^ (ref. 3). ECF transporters consist of two identical or highly similar cytoplasmic ATPases, EcfA and EcfA′, and two transmembrane subunits, EcfT and the S-component^1^,^2^,^4^. In contrast to classical ABC importers ECF transporters do not make use of additional substrate-binding domains or proteins. The membrane-embedded S-component serves as both the substrate-binding and the substrate-translocation domain. The other three subunits (EcfA, EcfA′ and EcfT) together form the ECF module that energizes substrate transport by the S-component^4^,^5^,^6^.

Based on the genomic location of the genes encoding the transporter subunits, ECF transporters have been categorized into two groups. Group I consists of dedicated transporters, in which the ECF module interacts with a single S-component, whereas group II ECF transporters share their ECF module with multiple S-components of different substrate specificities^2^. S-components from group II transporters can be released from the ECF module, and compete with each other for binding to the module^7^,^8^,^9^.

Several crystal structures of solitary S-components from a variety of organisms have been solved^10^,^11^,^12^,^13^,^14^. Despite little sequence identity (∼15%) the S-components for different substrates share a similar fold with a core of six membrane-embedded α-helices. In addition, three crystal structures of complete group II ECF transporters from *Lactobacillus brevis* (*L. brevis*) have been solved, all containing the same ECF module and a different S-component: ECF–FolT, ECF–HmpT and ECF–PanT^5^,^6^,^15^. The structures have provided the first glimpse of the mechanism of transport and the shared utilization of the ECF module by group II transporters.

Based on the structures, as well as mutational, accessibility and cross-linking studies on EcfT and S-components from different organisms^4^,^14^,^15^,^16^, a toppling mechanism of transport has been proposed, in which the S-component rotates in the lipid bilayer to expose its binding site alternately to the exterior (helices oriented as membrane-spanning) or the interior (toppled orientation with helices parallel to the membrane). The latter state was captured in the structures of the full complexes and is referred to as post-translocation state^15^, in which the substrate has been released into the cytosol. ATP binding in the ATPase subunits is thought to cause a conformational change in EcfT, which allows the S-component to topple back to the outward facing state. In group II ECF transporters ATP binding and hydrolysis also leads to release of the substrate-free S-component from the ECF module and allows for competition among different substrate-bound S-components for the same ECF module^7^,^8^,^9^.

Here we report crystal structures of the group II ECF transporter for folate from *Lactobacillus delbrueckii* (*L. delbrueckii*) in the *apo*-state and in complex with the slowly hydrolysable ATP analogue AMP–PNP. In addition, we present a structure of solitary FolT from the same organism in the folate-bound state. Comparison of the structures of FolT in the complex and in isolation reveals how solitary FolT captures substrate from the extracellular side, and how association with the ECF module is coupled to substrate release in the cytosol. We re-evaluate existing data in the light of new structures and provide a working model for the transport mechanism that can explain a crucial observation that led to the discovery of ECF transporters: the more effective competition of S-components in the substrate-bound state for the ECF module than proteins in the substrate-free state.

## Results

### S-components for folate in *L. delbrueckii*

The genome of *L. delbrueckii* encodes two FolT homologues, which we named FolT1 and FolT2. They share 93% sequence identity and have likely arisen from a recent gene duplication. FolT1 and FolT2 share only between 30 and 33% identity with the characterized FolT proteins from *L. brevis*, *Enterococcus faecalis* (*E. faecalis*) and *Lactobacillus casei* (*L. casei*) (Supplementary Fig. 1)^5^,^14^,^17^. Purified FolT1 and FolT2 in detergent solution bound folate with high affinity, with dissociation constants of 1.0±0.24 and 3.1±1.4 nM, respectively (s.d.'s from three independent experiments, Supplementary Fig. 2). The affinities of FolT1 and FolT2 for folate are higher than the affinities determined previously for the FolT proteins from *E. faecalis* (29.8 nM)^14^ and *L. casei* (9 nM)^17^. FolT1 and FolT2 differ in only 12 residues, none of which interact with folate, with the possible exception of residue 122 (see below), which is a tyrosine in FolT1 and an asparagine in FolT2. The FolT1 mutant Y122N binds folate with a dissociation constant of 2.5±1.2 nM (Supplementary Fig. 3), indicating that the tyrosine contributes to a slightly higher affinity, although the differences are small.

### Crystal structure of folate-bound FolT1

**Figure 1:**
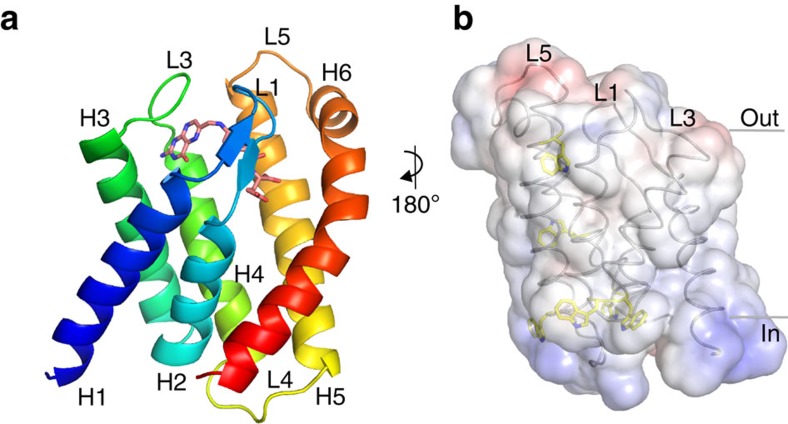
Crystal structure of folate-bound FolT1. (**a**) Cartoon representation of FolT1 coloured from blue (N terminus) to red (C terminus), with folate shown in stick representation. H1-6 indicates α-helices 1-6. The carbon atoms of folate are shown in deep salmon, the oxygen and nitrogen atoms are shown in red and blue, respectively. The colour coding for oxygen and nitrogen atoms remained throughout the article. The membrane orientation was derived from the hydrophobicity of the surface, the positive inside rule and the location of aromatic residues shown in **b**, in which the transparent surface is coloured according to the surface electrostatic potential (negative potential in red and positive potential in blue), and tryptophan residues are shown in sticks with their carbon atoms coloured yellow. The bottom of the protein faces the cytoplasm, while the top faces the extracellular side of the membrane.

We determined the crystal structure of folate-bound FolT1 at 3.0 Å resolution (Table 1). FolT1 consists of six membrane-embedded α-helices (Fig. 1a,b), which have a similar structure as other solitary S-components with specificities for various substrates (RibU^10^, ThiT^11^, BioY^12^, NikM2 (ref. 13), FolT^14^). Structurally, FolT1 is most closely related to FolT from *E. faecalis* with which it shares 30% sequence identity.

Based on the hydrophobicity of the helical segments and the positive-inside rule^18^ solitary FolT1 is predicted to be oriented in the membrane with the N- and C-terminal ends and loops L2 and L4 located in the cytoplasm, and loops L1, L3 and L5 facing the extracellular environment (Fig. 1b). However, deduction of the membrane orientation from the structure is difficult in this case because we know that the FolT protein can topple over in the context of the complete ECF transporter (see below). Nonetheless, it is plausible that solitary FolT can adopt the orientation shown in Fig. 1a as it is in agreement with molecular dynamics simulations that revealed a similar orientation for the solitary S-component ThiT in a lipid bilayer^19^.

Well-defined non-protein electron density inside the α-helical core was assigned to the folate molecule (Supplementary Fig. 4). The occluded folate-binding site is lined by all six membrane α-helices and is located close to the extracellular side of the membrane (orientation as in Fig. 1a), where it is shielded from the exterior by the L1 loop connecting helices 1 and 2, and the L3 loop between helices 3 and 4. The substrate-binding pocket has a volume of about 1,400 Å^3^, which leaves a vast amount of space for extensions on the folate molecule such as a polyglutamate tail. A detailed description of the interactions is provided in the supplement, but it is important to note that loop L1 and loop L3 provide many of the interactions with the folate molecule. The pterin moiety of the folate molecule forms a network of strong interactions with residues from helices 3 and 4, and the connecting loop L3 (Supplementary Fig. 4c), while the aminobenzoate and glutamate moieties interact with residues from helices 5 and 6, and loop L1 (Supplementary Fig. 4d).

### Transport activity of ECF–FolT1 and ECF–FolT2

**Figure 2:**
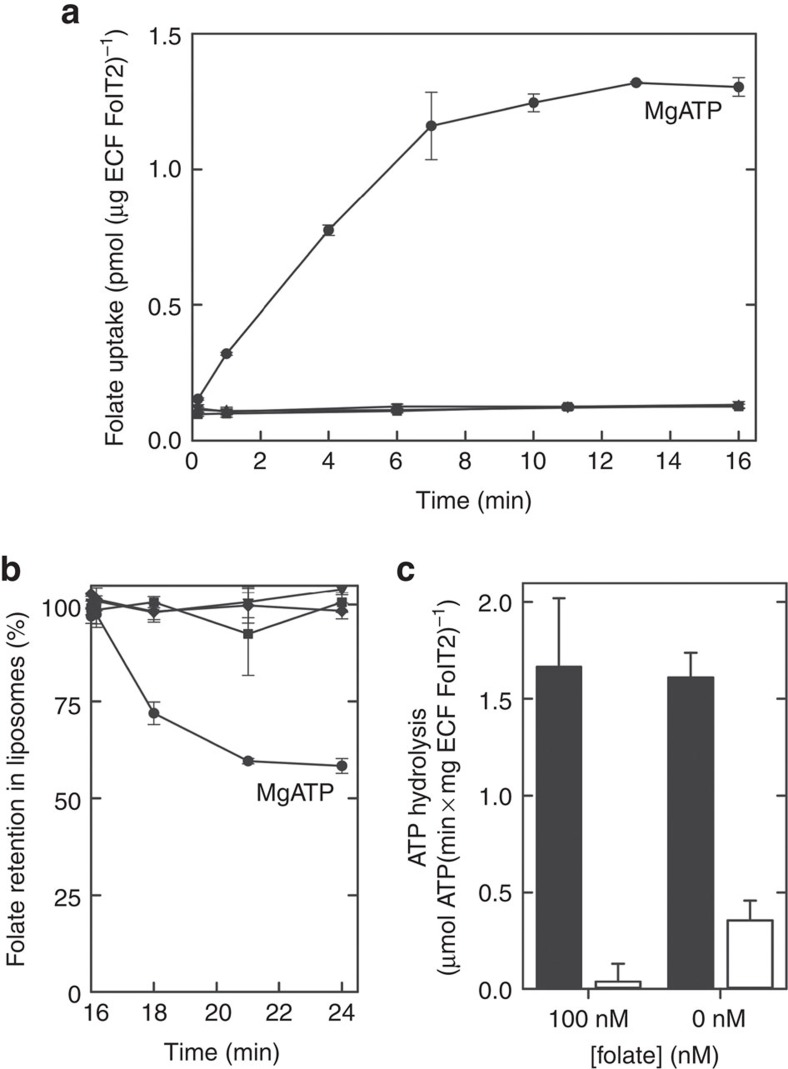
Transport and ATPase activity of ECF–FolT2. (**a**) Transport activity of ECF–FolT2 in proteoliposomes loaded with 5 mM of MgATP (circles), 5 mM MgADP (inverted triangles), 5 mM MgAMP–PNP (triangles) or 5 mM Na_2_ATP plus 5 mM EDTA (squares). (**b**) Efflux activity of ECF–FolT2 from proteoliposomes loaded with 5 mM of MgATP, after accumulation of radiolabelled folate for 16 min. At *t*=16 min, 5 mM of MgATP (circles), 5 mM MgADP (inverted triangles), 5 mM Na_2_ATP plus 5 mM EDTA (squares) or 100 μM non-radiolabelled folate (diamonds) was added to the reactions. (**c**) ATPase activity of ECF–FolT2 reconstituted in proteoliposomes (black bars) and background ATPase activity by empty liposomes (white bars). When indicated, folate was present both in the lumen of the liposomes and in the environment. The error bars show the s.d.'s from three independent measurements.

FolT1 and FolT2 interact with the same ECF module to assemble into full ECF transporters. We purified the ECF–FolT1 and ECF–FolT2 complexes, reconstituted them into proteoliposomes and performed transport assays with radiolabelled folate (ECF–FolT2: Fig. 2a, ECF–FolT1: Supplementary Fig. 5). Both ECF transporters mediated association of radiolabelled folate with the proteoliposomes in the presence of luminal MgATP, but not in the presence of MgADP. Although these experiments suggest that the ECF transporters translocate folate across the membrane into the lumen, they are not conclusive. The reason for the ambiguity is that there are apparently more protein molecules present in the liposomes than folate molecules associating with them. Therefore it cannot be excluded that MgATP-dependent binding of folate to the proteins might have occurred, without translocation. In the case of ECF–FolT2 there was an almost seven-fold excess of protein complexes (8.7 pmol, assuming a reconstitution efficiency of 100%) over folate molecules associated with the liposomes (1.3 pmol). Transport assays reported previously using other purified and reconstituted ECF complexes from *Lactococcus lactis* (*L. lactis*) and *L. brevis*, showed similar ambiguities, although they were not discussed. In these cases, we calculated protein to substrate excesses of 12.7-fold (ECF–NiaX^20^), 29.0-fold (ECF–RibU^20^) and 627-fold (ECF–FolT^5^).

To unambiguously show that ECF–FolT2 could transport folate across the membrane, we made use of the observation that the orientation of membrane proteins in liposomes is often scrambled during the reconstitution procedure: liposomes contain both right-side-out- and inside-out-oriented protein complexes. If the ECF complexes can catalyse transport rather than just binding, the inside-out-oriented complexes should allow export of luminal folate from the proteoliposomes in the presence of external MgATP. To test this prediction, we performed a folate uptake experiment as described above until a steady-state level of folate association was reached (after 16 min, Fig. 2b), and then added external MgATP or MgADP to the liposomes. Efflux of the radiolabelled folate occurred on addition of MgATP, but not on addition of MgADP, which shows that ECF transporters indeed translocate their substrates across the membrane and use an ATP-dependent mechanism. As a further control, we added a 1000-fold excess of non-radiolabelled folate to the proteoliposomes after the steady-state level of folate association had been reached. The excess folate did not chase off the accumulated radiolabel, which again is consistent with transport rather than binding.

To test whether ATP binding is sufficient for transport or ATP hydrolysis is required, we filled the proteoliposomes with ATP and EDTA (in the absence of Mg^2+^) or with the slowly hydrolysable ATP analogue AMP–PNP (in the presence of Mg^2+^) (Fig. 2a). In neither condition folate transport occurred, indicating that ECF–FolT mediates folate translocation only under ATP hydrolysing conditions.

Beside folate transport, we also measured the ATPase activity of ECF–FolT2 in the proteoliposomes, both in the presence and absence of 100 nM of folate (Fig. 2c). The presence of folate did not significantly affect ATPase activity indicating poor coupling between transport and nucleotide hydrolysis. High futile ATPases activity has also been observed for the group I transporter BioMNY^16^. Comparison of the rate of ATP hydrolysis with the rate of folate transport corroborates that there is poor coupling, as the ATPase rate is more than four orders of magnitude higher than the transport rate. Apparently futile ATP hydrolysis is an inherent characteristic of the ECF transporter.

### Crystal structures of ECF–FolT2 complexes

**Figure 3:**
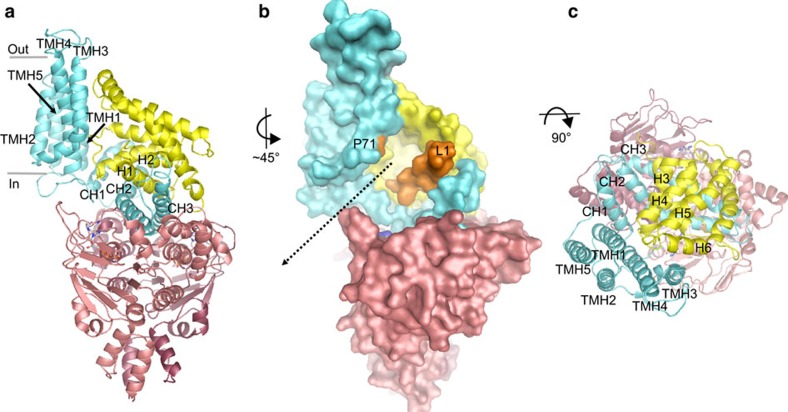
Crystal structure of AMP–PNP bound ECF–FolT2. (**a**) Cartoon representation of ECF–FolT2 viewed from the plane of the membrane, with EcfA and EcfA′ coloured in two shades of red, EcfT in cyan and FolT2 in yellow. AMP–PNP molecules are shown in sticks representation, with the carbon and phosphor atoms coloured grey and orange, respectively. The transmembrane helices of EcfT are indicated by TMH1-5 and the three coupling helices by CH1-3. (**b**) Surface representation using the subunit colours from **a**. Loop L1 of FolT2 and proline 71 (P71) in TMH3 of EcfT are coloured orange. The pathway leading from the open folate-binding cavity to the cytosol is indicated by the dashed arrow. (**c**) View along an axis perpendicular to the membrane.

We determined crystal structures of the ECF–FolT2 complex from *L. delbrueckii* in the *apo* state (no nucleotides or folate) and in an AMP–PNP-bound state at 3.0 and 3.3 Å resolution, respectively (Table 1). The overall structures of the two complexes are shown in Fig. 3 and Supplementary Fig. 6. Although both structures globally resemble the structures of the ECF transporters from *L. brevis*^5^,^6^,^15^, the corresponding subunits share only between 30 and 50% sequence identity. Importantly, the comparison of the structures of solitary FolT1 and the full ECF–FolT2 from a single organism now allows insight into the conformational transitions that take place during transport that cannot be obtained from the comparison of proteins with low sequence similarity from different organisms.

In both structures of ECF–FolT2, the ATPases EcfA and EcfA′ are in an open conformation with the subunits separated thus leaving the two ATP hydrolysis sites incomplete. In the AMP–PNP bound structure, EcfA and EcfA′ each bind a molecule of the nucleotide, but binding does not lead to closure of the ATPase dimer (see below). EcfT can be divided into two domains: the transmembrane domain formed by five transmembrane helices (TMH1-5), and the coupling domain formed by the three coupling helices (CH1-3). The cytosolic side (bottom) of the coupling domain interacts with the ATPase subunits, whereas the membrane side (top) binds to the S-component. The membrane domains of EcfT are in different conformations in the two structures (see below). Compared with the putative membrane orientation of solitary FolT1 (Fig. 1a), FolT2 has toppled over and the helices lie roughly parallel to the membrane plane (Fig. 3a,c). Because the AMP–PNP and *apo* complexes have similar structures, we will focus our further discussion on the AMP–PNP-bound structure, and consider the *apo* complex only when it differs from the AMP–PNP structure.

### Comparison of solitary FolT1 with FolT2 in the complexes

**Figure 4:**
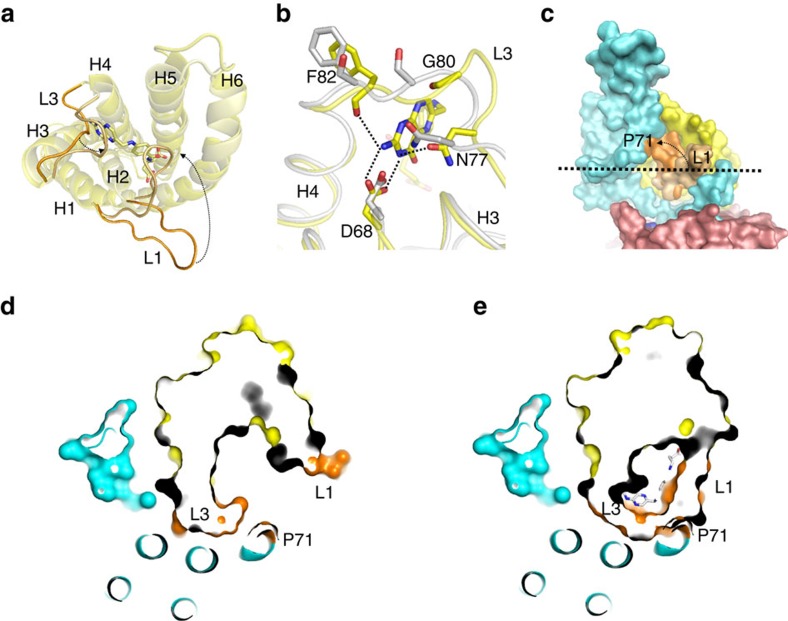
Comparison of folate-bound FolT1 and substrate-free FolT2. (**a**) Structural alignment of folate-bound FolT1 (pale yellow) and substrate-free FolT2 (bright yellow). The viewpoint is from the extracytoplasmic side of the membrane. The carbon atoms of folate are shown in pale yellow and loops L1 and L3 are coloured in pale and bright orange for FolT1 and FolT2, respectively. (**b**) Repositioning of loop L3 of FolT2 (grey) compared with FolT1 (yellow) disrupts the interactions with folate, of which the carbon atoms are coloured yellow. Binding-site residues are indicated using the one-letter code for amino acids. (**c**) Surface representation showing how loop L1 (orange) of substrate-bound FolT1 would clash with P71 (orange) of EcfT when forming a complex with the ECF module. Loop L1 of FolT2 is shown in pale orange for comparison. The dashed arrow shows the movement of loop L1 between FolT2 and substrate-bound FolT1. (**d**) Slice-through representation of ECF–FolT2 at the level of the dashed line in **c**. FolT2 is shown in surface representation with loops L1 and L3 in orange, and P71 of EcfT in orange surface representation. (**e**) The same slice as shown in **d** but with substrate-bound FolT1 replacing FolT2, with the carbon atoms of folate shown in grey. Loops L1 and L3 would clash with P71.

The FolT2 proteins in the AMP–PNP-bound and *apo* complexes do not have folate bound. Nonetheless, the structure of substrate-free FolT2 is very similar to the structure of solitary, folate-bound FolT1 with root mean squared deviation values of 1.35 Å (FolT1 compared with FolT2 from the AMP–PNP-bound structure) and 1.25 Å (FolT1 compared with FolT2 from the *apo* structure). All α-helical segments and cytoplasmic loops are in almost identical conformations. The only differences are the conformations of the L1, L3 and L5 loops (Fig. 4a). In FolT1, L1 and L3 cover the folate-binding pocket and provide multiple interactions with the substrate, whereas they have moved away in the substrate-free state, creating a wide opening. On the movements of the L1 and L3 loops, the interactions with folate are lost and the high-affinity binding site is destroyed (Fig. 4b discussed below). Loop L5 also adopts a slightly different conformation but this displacement does not affect the folate-binding site directly (Fig. 4a). The loss of the high-affinity binding site for folate in the complex explains why no electron density for folate was found in the structure of the AMP–PNP-bound complex even though a high concentration of the substrate was added during crystallization. Consistently, biochemical characterization of ECF–RibU has shown that riboflavin could bind only to solitary RibU, but not to the full complex^8^. The structures also explain how folate is released into the cytoplasm, because the L1 and L3 loops that occluded the binding site in the solitary S-component are now moved and allow passage of the substrate towards the cytoplasm.

The structural resemblance of FolT1 and FolT2 indicates that only small conformational changes take place in S-component on substrate binding or release. This notion is consistent with EPR and pre-steady-state fluorescence experiments on the thiamin binding to the S-component ThiT^19^ that showed minor conformational changes on binding and very rapid association kinetics, close to the diffusion limit. From the outward-occluded state of solitary FolT1 (orientation shown in Fig. 1), small movements of loops L1 and L3 would be sufficient to open access to the substrate-binding site to the extracellular side of the membrane. High-affinity folate binding from the outside has indeed been demonstrated in whole cell assays^17^. The small conformational changes combined with high-affinity substrate binding make solitary S-component efficient scavengers for scarce and precious substrates in the environment of the bacteria.

### Interaction of FolT with EcfT triggers substrate release

**Figure 5:**
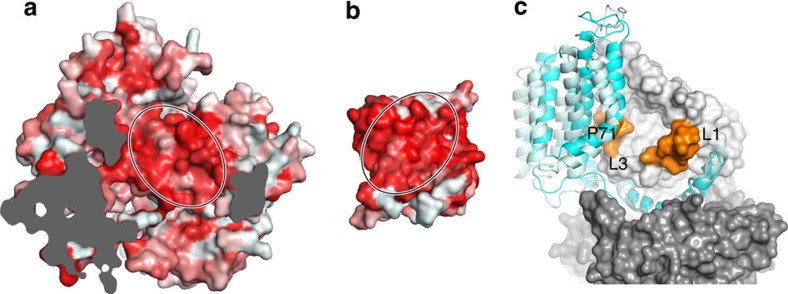
Sites of interaction between EcfT and FolT2. (**a**) Slice-through of the ECF module in surface representation viewed from the extracellular side of the membrane. FolT2 has been deleted to show the hydrophobic platform on top of the coupling domain of EcfT, indicated by the oval. Colouring according to hydrophobicity, from red (hydrophobic) to grey (hydrophilic). (**b**) Surface of FolT2 interacting with the surface shown in **a**, using the same colour-coding. (**c**) Structural flexibility in the EcfT subunit. The *apo* and AMP–PNP-bound structures were superimposed by structural alignment of the ATPase subunits (dark grey surface representation). The resulting positions of the EcfT subunits are shown in cartoon representation with a viewpoint from the membrane plane. The coupling domains with coupling helices CH1-3 are in almost identical positions in the two complexes, but the membrane domains differ. The membrane domain in the *apo*-structure (grey) has hinged away compared with the AMP–PNP-bound structure (cyan). Proline 71 on TMH3 of EcfT as well as loops L1 and L3 in FolT2 are shown in orange.

The high-affinity folate-binding site is destroyed in the structures of the ECF–FolT2 complex, which allows for accumulation of substrate in the cytoplasm, and likely results in higher off-rates than in the solitary S-components, leading to physiologically relevant transport rates. The free energy required to destroy the binding site must be provided by the interaction energy liberated upon docking of FolT to the ECF module. The coupling domain of EcfT provides the main surface for interaction with FolT2, with the three coupling helices forming a hydrophobic platform that binds an equally hydrophobic surface on helices 1 and 3 of FolT2 (Fig. 5a,b). Two alanines of the well-conserved AxxxA motif in helix 1 of FolT, which have been shown to be crucial for the interaction of other S-components with the ECF module^11^,^15^, are part of the interaction surface. The shapes of the two surfaces are highly complementary creating a tight fit.

The structure of the surface interacting with EcfT is identical in substrate-free FolT2 and folate-bound FolT1, which raises the question how the folate binding site is disrupted in the complex. The structures show that the association of FolT to EcfT via the hydrophobic interface allosterically disrupts the folate-binding site. The conformations of loops L1 and L3 in folate-bound FolT1 are incompatible with binding to the ECF module, because they would clash with TMH3 of the membrane domain of EcfT at the position of the conserved P71 (Fig. 4c–e). This proline also introduces a kink in TMH3, which directs the N-terminal end of the helix away from the S-component, allowing a wide passageway from the folate-binding pocket to the cytoplasm (compare Figs 3b and 4c–e). The steric clash with TMH3 shows that toppling of FolT can occur only if simultaneous displacement of loops L1 and L3 takes place, which results in loss of the folate-binding site. Thus, high-affinity binding of folate to FolT and interaction of EcfT with FolT are mutually exclusive.

The displacement of loops L1 and L3 in the toppled state is stabilized by interactions with EcfT. As will be discussed below, these interactions are probably much weaker than the interaction between the hydrophobic surfaces of FolT2 and the coupling domain of EcfT. The position of the L1 loop in the complexes is stabilized by a hydrogen bond between the backbone carbonyl group of G35 and the highly conserved R196 (the AMP–PNP structure) or N189 (in the *apo* structure) in the coupling domain of EcfT. The L3 loop interacts with a conserved serine- and threonine-rich stretch (STφφTφTT, in which φ is a hydrophobic residue) in TMH4 of EcfT. The hydroxyl groups of the side chains of S123 and T127 from this motif form hydrogen bonds with the backbones of G76 and G80 in the L3 loop (Supplementary Fig. 7a), leading to a conformation of the L3 loop that cannot form the backbone interactions with the folate molecule found in FolT1, and forcing the side chain of N77 into the position of the pterin ring (Fig. 4b).

Besides loops L1 and L3, the C-terminal end of H5 from FolT2 also interacts with EcfT. The partial negative charge from the helix dipole binds to the positive charge of conserved R115 in TMH4 in the membrane domain of EcfT (Supplementary Fig. 7b). The interactions between EcfT and loops L1, L3 and H5 are not dependent on the amino-acid sequence of the S-component because either backbone groups (loops L1 and L3) or the negative charge from a helix dipole (H5) are involved. The sequence-independence of the interactions explains how S-components for different substrates can topple along and bind to the same ECF module.

### AMP–PNP-binding does not lead to a closed conformation

**Figure 6:**
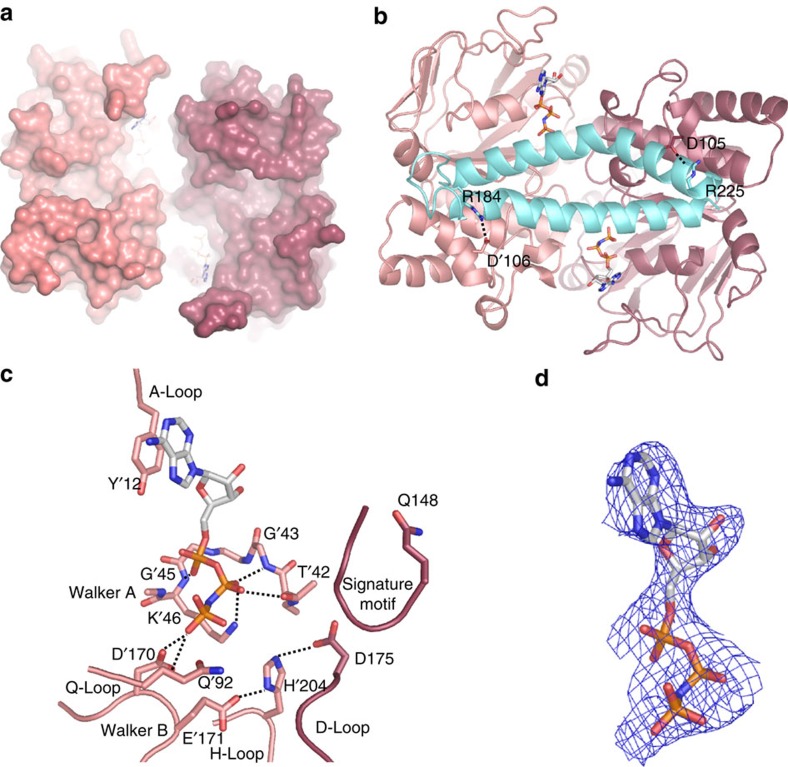
AMP–PNP binding to the EcfAA′ heterodimer. (**a**) Surface representation AMP–PNP-bound EcfAA′ heterodimer coloured as in Fig. 3 with the AMP–PNP molecules in stick representation. The viewpoint is from the extracytoplasmic side (**b**) Cartoon representation of the AMP–PNP-bound EcfAA′ heterodimer and the coupling helices CH2 and CH3 of EcfT. The conserved arginines at the C-terminal end of the coupling helices, and the interacting aspartates in the EcfAA′ heterodimer are shown in sticks. The interactions are indicated by the dashed lines. (**c**) AMP–PNP-binding site on EcfA′ (coloured light red). The conserved motifs found in ABC transporter ATPases are indicated. (**d**) 2Fo–Fc electron density at 2σ in blue contouring AMP–PNP (same viewpoint as in **c**).

Two patches of well-defined electron density were observed in EcfA and EcfA′ of the complex crystallized in the presence of Mg–AMP–PNP and were assigned to the nucleotide molecules (Fig. 6). The two AMP–PNP molecules interact with conserved motifs that are essential for ATP binding and hydrolysis in other ABC transporters^21^. All the important motifs are present in both subunits, which share 35% sequence identity (Supplementary Fig. 8). Even though AMP–PNP molecules are bound to both subunits, binding does not lead to the closed conformation of the ATPases. The AMP–PNP molecules interact only with motifs from a single ATPase subunit, and are separated from the signature motifs (LSGGQ) on the other subunit (Fig. 6c). Therefore the ATP hydrolysis sites are still incomplete. A detailed description of the AMP–PNP-binding sites is provided in the supplement.

### Structural flexibility in the membrane domain of EcfT

Although the structures of the *apo* and AMP–PNP-bound complexes are very similar overall, the relative orientation of the transmembrane and coupling domain of EcfT is different (Fig. 5c). The coupling domains of the two complexes are in identical positions relative to the two ATPases and FolT. Consequently, the hydrophobic interface that interacts with FolT (Fig. 5a) and the interface interacting with the ATPase subunits (Fig. 6b) are both intact in the two structures. In contrast, the transmembrane domains are in different orientations. In the AMP–PNP structure it is packed tightly against the FolT subunit and interacts with the loops L3 and the C terminus of H5, but in the *apo* structure it has hinged away and the two membrane proteins interact less tightly (Fig. 5c). It is likely that the structural flexibility of the membrane domain serves two functions. First, it allows toppling, which may require structurally different parts of FolT (loops L1, L3 and L5) to slide along the membrane part of EcfT. Second, EcfT has to be sufficiently flexible to allow the binding of different S-components with different sequences and structures in group II ECF transporters, which use the same ECF module^15^. The structural flexibility of the membrane domain of EcfT also suggests that the interactions with loop L3 and H5 from FolT are relatively weak, in contrast to the strong hydrophobic and van der Waals interactions between the rigid coupling domain of EcfT and FolT.

## Discussion

**Figure 7:**
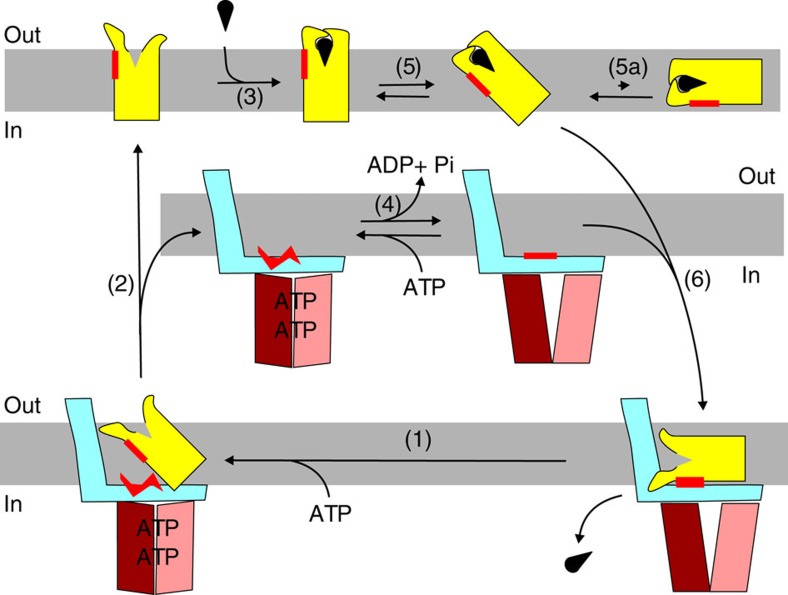
Working model for the transport mechanism of group II ECF transporters. Colours of the subunits as in Fig. 3. Starting with the complex trapped in the post-translocation state, binding of ATP is needed to release the empty S-component by disruption of the hydrophobic interface (red) (step (1)). The S-component will reorient to the outward-facing state (2) and can bind substrate on the extracellular side of the membrane (3). ATP hydrolysis in the ECF module regenerates the binding platform for the S-component (4). Possibly futile ATP hydrolysis takes place in this stage. The substrate-bound S-component can topple over in the membrane possibly aided by the vicinity of the ECF module (5). The toppled S-component binds to the ECF module via the complementary hydrophobic surfaces, coloured in red (6). Binding of the S-component to the ECF module forces the disruption of the substrate-binding site and release of the substrate into the cytoplasm.

Based on the work presented here and a wealth of data on various ECF transporters dating back to the 1970s, we tentatively provide a scheme for the transport cycle consistent with the available experimental data (Fig. 7). There are important differences compared with previously published models, which we will discuss below.

In all structures of complete ECF transporter complexes^5^,^6^,^15^, the S-components are trapped in an inward-facing post-translocation state, in which they interact tightly with the coupling domain of EcfT via complementary hydrophobic surfaces. To escape from this state the interaction interface must be disrupted, which requires input of energy. Presumably the formation of a closed EcfA–EcfA′ dimer by the binding of ATP, deforms the coupling helices from the EcfT subunit, thus destroying the hydrophobic interaction surface (step (1) in Fig. 7). ATP binding to the group II ECF–RibU transporter from *Listeria monocytogenes*^8^ has indeed been shown to drive the release of the S-component from the ECF module. The solitary S-component in the absence of substrate reorients to expose the empty binding site to the outside of the cell, ready to bind a new substrate (step (2) in Fig. 7). The high affinity of the S-components for their substrates subsequently allows for efficient scavenging from the environment (step (3) in Fig. 7). For group I ECF transporters, it has been suggested that the reorientation step does not lead to complete dissociation of the S-component from the ECF module^16^. Although it is possible that group I transporters indeed use a different mechanism, it is noteworthy that the experiments indicating the lack of dissociation were conducted in a nanodisc environment, which may prevent the detection of dissociation because of physical confinement.

Our structural work shows that the binding of AMP–PNP to ECF–FolT2 is not sufficient to form the closed EcfA–EcfA′ dimer. The lack of closure contrasts with the observed full closure when AMP–PNP binds to a partial complex of the ATPase subunits without the membrane subunits^8^. Apparently, closure is more difficult in the context of the complete complex. In other ABC transporters the binding of AMP–PNP sometimes led to closed ATPase subunits (for example, in the maltose transporter^22^,^23^), but in other cases left the ATPases in an open conformation^24^.

ATP is hydrolysed when the transport cycle proceeds. The requirement of full ATP hydrolysis for transport in lipid bilayers is obvious from the transport experiments presented in Fig. 2. The high basal ATPase activity of the ECF–FolT2 transporter when reconstituted in liposomes suggests that continuous ATP turnover may take place in the ECF module (step (4) in Fig. 7). Futile ATP consumption may be an acceptable trade-off to ensure capturing as much as possible of scarcely available nutrients. The high basal ATPase activity probably can only be sustained in the cell if low amounts of the ECF module are present. Indeed, the levels of the ECF module are usually much lower than the levels of S-components^2^.

In previously published models it has been postulated that toppling of the S-component from the outward- to the inward-facing orientation requires strict coupling to ATP hydrolysis^8^,^16^. In contrast, we hypothesize that solitary substrate-loaded S-components have an intrinsic ability to topple over in the lipid bilayer (step (5) in Fig. 7). Such toppling may be possible because the substrate-bound S-components form compact structures without exposed charged residues on the extracellular face. The toppled proteins will be trapped by the hydrophobic binding platform of EcfT, which consequently destroys the high-affinity binding site and leads to substrate release in the cytoplasm (step (6) in Fig. 7). A strong indication that spontaneous toppling can indeed take place comes from the observation that some S-components can mediate substrate transport in the absence of an ECF module^25^,^26^,^27^,^28^.

Transport by solitary S-components is incompatible with strict coupling between ATP hydrolysis in the ECF module and toppling of the substrate-loaded S-component. Moreover, there is no conclusive experimental evidence for such coupling. For the group I transporter BioMNY, it has been shown that ATP hydrolysis is required for substrate release, but the data did not reveal the sequence of events^16^. ATP hydrolysis may take place in the absence of the S-component, only to reset the ECF module to the state that can interact tightly with the S-component again (steps (4) and (6) in Fig. 7). Even though strict coupling between ATP hydrolysis and toppling may not be necessary, it is possible that continuous ATP hydrolysis by the ECF module (step (4) in Fig. 7) facilitates toppling by distorting the lipid bilayer in the vicinity of the S-component.

In the 1970s, shared use of the ECF module by different S-components in group II transporters had already been proposed based on *in vivo* uptake experiments^7^. These experiments showed that substrate-loaded S-components compete more efficiently for the limited number of ECF modules than the substrate-free proteins. Recently these experiments have been reproduced using recombinant proteins produced in *Escherichia coli* (*E. coli*)^9^. In the light of the structures presented here it is very unlikely that tighter binding of substrate-loaded than substrate-free S-components to the ECF module would cause the competitive advantage. In fact, substrate-bound FolT seems to be unable to interact with the ECF module because of steric clashes between loops L1 and L3, and the membrane domain of EcfT. To explain the competition experiments, we hypothesize that substrate-loaded S-components do not bind more tightly to the ECF module than the substrate-free proteins, but they can reach the binding platform more efficiently. The observed competition is then caused by differences in the kinetics of toppling. Substrate-free S-components cannot topple over as easily because their hydrophilic loops L1 and L3 are fully exposed. Therefore they are unable to reach the hydrophobic interaction platform of EcfT, even though they would bind to it very tightly if they could.

Importantly, the mechanism of competition described above does not require the existence of a stable state in which the ECF module is in complex with a substrate-loaded S-component. The existence of such a state was postulated when structures of the same S-component in the substrate-bound and *apo*-conformation were not yet known^8^,^16^. In the work on the group I transporter ECF–BioY, the authors detected biotin binding to the solitary BioY, but not to the full complex. The authors postulate that an inaccessible (occluded) binding site exists in the complex^16^. Based on the structural data presented here and published previously, we explain the lack of binding by the disruption of the binding site, and consequent extrusion of biotin on the cytoplasmic side. The scheme presented here does not require the postulation of an occluded state, yet can explain the ensemble of experimental data, and therefore is more parsimonious than previous models.

For the group II transporter ECF–RibU the existence of a stable riboflavin-bound full complex has also been proposed. A mutant that could bind but not hydrolyse ATP readily exchanged its S-component RibU with a fluorescently labelled solitary RibU molecule in the presence of ATP in detergent solution^8^. Although this experiment elegantly supports the ‘catch-and-release' mechanism of S-components, it does not require that the substrate riboflavin is bound to RibU in the complex. Even if riboflavin would bind to the full complex, the affinity might be much lower than for solitary RibU, consistent with the model proposed here.

The working model for the mechanism of transport presented in Fig. 7 accounts for the observed high basal ATPase activity, the competition of different S-components for the same ECF module, the release of S-component from the ECF module during turnover and substrate release from the S-component during toppling. This model will be used as handle for further experiments to study the toppling mechanism.

## Methods

### Cloning

The genes encoding EcfA, EcfA′ and EcfT of *L. delbrueckii* subsp. *bulgaricus* are annotated as *cbiO* (both for LDB_RS01805, *ecfA* and LDB_RS01810 *ecfA′*) and *cbiQ* (LDB_RS01815, *ecfT*). The ECF module operon was cloned downstream of the first arabinose inducible promoter of the p2BAD vector^29^ between the *Bsp*E1 and *Bgl*II sites, in-frame with a sequence coding for a N-terminal His_10_-tag and TEV cleavage site before EcfA. *folT1* (LDB_RS07025) or *folT*2 (LDB_RS07030) was cloned downstream of the second promoter, between the *Xba*I and *Xho*I sites in-frame with a sequence coding for a C-terminal STREPII tag (WSHPQFEK). The p2BAD His_10_-ECF–FolT1/2-STREPII plasmid was transformed into Ca^2+^-competent cells of the *E. coli* strain MC1061.

Solitary *folT1* and *folT2* were cloned in the pREnHis vector, which contains the sequence encoding for a N-terminal His_8_-tag. Subsequently, the vectors were converted into *L. lactis* expression vectors using the vector backbone exchange protocol^30^. The resulting pNZnHisFolT1/2 vectors were transformed into electrocompetent cells of the *L. lactis* strain NZ9000^31^. Mutations in the FolT1/2 sequence were introduced using the primers given in Supplementary Table 1.

### Expression and membrane vesicle preparation

Expression of ECF–FolT1/2 was performed in a 5-l flask containing 2 l of yeast trypton medium (8 g l^−1^ Bacto trypton, 5 g l^−1^ Bacto yeast extract, 2.5 g l^−1^ NaCl), supplemented with 2.5 mM potassium phosphate (KPi), pH 7.0, 0.5% glycerol and 100 μg ml^−1^ ampicillin. The *E. coli* MC1061 cells with p2BAD His_10_-ECF–FolT1/2-STREPII were grown at 37 °C, 200 r.p.m. to an OD_600_ of 0.8, after which the temperature was reduced to 25 °C. After allowing the cultures to cool down for 20 min, expression was induced by addition of 1.0 × 10^−2^% arabinose. After 3 h of expression the cells were collected by centrifugation (15 min, 7,446*g*, 4 °C), washed in buffer A (50 mM KPi, pH 7.5) and resuspended in the buffer B (50 mM KPi, pH 7.5, 10% glycerol). Membrane vesicles were either prepared immediately, or the resuspended cells were stored at −80 °C after flash freezing in liquid nitrogen.

For folate-binding assays substrate-free FolT1 and FolT2 were produced in *L. lactis* cells grown semi-anaerobically in chemically defined medium^32^ without folate, and supplemented with 2.0% (w/v) glucose, 5 μg ml^−1^ chloramphenicol, in a 2l fermenter at 30 °C and pH 6.5. The cell culture was induced at an OD_600_ of ∼1.5 by addition of 0.1% (v/v) culture supernatant of a Nisin A-producing strain^31^. For crystallization FolT1 and FolT2 were produced in *L. lactis* cells grown in M17 broth (Difco), supplemented with 2.0% (w/v) glucose, 5 μg ml^−1^ chloramphenicol, in a 1l bottle. In this case the cell culture was induced at OD_600_ ∼0.8 by addition of 0.2% (v/v) culture supernatant of the Nisin A-producing strain. After 3 h of expression, the cell cultures were collected and washed as described above for the expression of ECF–FolT2, but in 50 mM KPi pH7.0 and 10% glycerol.

Before membrane vesicle preparation, 1 mM MgSO_4_ and ∼50–100 μg ml^−1^ DNase were added to the cells. The cells were lysed by high-pressure disruption (Constant Cell Disruption System Ltd, UK, two passages at 25 kPsi for *E. coli* cells or at 39 kPsi for *L. lactis* cells, 5 °C) and cell debris was removed by low-speed centrifugation (30 min, 12,074*g*, 4 °C). Membrane vesicles were collected by ultracentrifugation (120 min, 193,727*g*, 4 °C), and resuspended in buffer B to a final volume of 5 ml per 1 l of cell culture. Subsequently, the membrane vesicles were aliquoted, flash frozen in liquid nitrogen and stored at −80 °C. The total protein concentration in the membrane vesicles was determined by Bradford Protein Assay (Bio-Rad).

### Protein purification

For the purification of ECF–FolT2, membrane vesicles were thawed rapidly and solubilized in buffer C (50 mM KPi, pH 7.5, 300 mM NaCl, 15 mM imidazole, 1% (w/v) *n*-dodecyl-β-D-maltopyranoside (DDM, Anatrace)) for 1 h at 4 °C, while gently rocking. Unsolubilized material was removed by centrifugation (20 min, 442,907*g*, 4 °C). The supernatant was incubated for 1 h at 4 °C under gently rocking with Ni^2+^-sepharose resin (column volume of 0.5 ml), which had been equilibrated with buffer D (50 mM KPi, pH 7.5, 300 mM NaCl, 50 mM imidazole, 0.05% (w/v) DDM). Subsequently, the suspension was poured into a 10-ml disposable column (Bio-Rad) and the flow through was collected. The column material was washed with 15 ml of buffer D. The ECF–FolT2 complex was eluted in three fractions of buffer E (50 mM KPi, pH 7.5, 300 mM NaCl, 500 mM imidazole, 0.05% (w/v) DDM) of 400, 750 and 500 μl, respectively. An amount of 1 mM of EDTA was added to the second elution fraction to remove co-eluted Ni^2+^ ions. Subsequently, the second elution fraction was purified by size-exclusion chromatography using a Superdex 200 10/300 gel filtration column (GE Healthcare), equilibrated with buffer F (50 mM KPi, pH 7.5, 150 mM NaCl, 0.05% (w/v) DDM) in case of purification for reconstitution or equilibrated with buffer G (20 mM Tris, pH 8.0, 150 mM NaCl, 0.05% (w/v) DDM) in case of purification for crystallization. After size-exclusion chromatography, the fractions containing the ECF–FolT2 complex were combined and used directly for reconstitution, or concentrated by the use of a Vivaspin 500 concentrating device with a molecular weight cutoff of 100 kDa (Sartorius stedim) to a final concentration of 5–8 mg ml^−1^ when used for crystallization.

FolT1 and FolT2 were purified using the same protocol, but with different buffers. Solubilization of the membrane vesicles was performed in buffer H (50 mM KPi, pH 7.0, 200 mM KCl, 1% (w/v) DDM), the Ni^2+^-sepharose resin was equilibrated and washed with buffer I (50 mM KPi, pH 7.0, 200 mM KCl, 50 mM imidazole, 0.35% (w/v) *n*-nonyl-β-D-glucopyranoside (NG, Anatrace)), the proteins were eluted from the Ni^2+^-sepharose column using buffer J (50 mM KPi, pH 7.0, 200 mM KCl, 500 mM imidazole, 0.35% (w/v) NG) and the Superdex 200 10/300 gel filtration column was equilibrated with buffer K (50 mM KPi, pH 7.0, 150 mM KCl, 0.35% (w/v) NG). After size-exclusion chromatography, the fractions containing FolT1 or FolT2 were combined and directly used for substrate-binding assays. When FolT1 was used for crystallization, the protein was incubated in the presence of 100 μM folate overnight at 4 °C under gently rocking and subsequently concentrated by the use of a Vivaspin 500 concentrating device with a molecular weight cutoff of 50 kDa (Sartorius stedim) to a final concentration of 8 mg ml^−1^.

### Crystallization and structure determination

Initial crystals of the apo ECF–FolT2 complex were found in the G8 condition (50 mM Tris, pH 7.5, 17% (v/v) PEG350 MME) of the MemGold2 HT-96 screen (Molecular Dimensions) using a Mosquito robot (TTP Labtech, UK) and diffracted up to 8 Å resolution. Optimizations using the Additive (HR2-428) and Detergent (HR2-408) screens of Hampton Research led to an optimized condition of 50 mM Tris, pH 7.5, 17% (v/v) PEG350 MME, 10 mM spermidine, 2% (w/v) NG, which yielded crystals diffracting up to 3.7 Å resolution. Multiple rounds of seeding in 24-well hanging-drop crystallization plates resulted in diamond-shaped crystals, which grew in clumps and diffracted up to 2.7 Å resolution. Using the apo ECF–FolT2 crystals for seeding, ECF–FolT2 in the presence of 10 mM MgCl_2_, 10 mM AMP–PNP and 1 μM folate crystallized in single diamond-shaped crystals diffracting up to 3.0 Å resolution. Diffraction data were collected at the European Synchrotron Radiation Facility (ESRF) at beamline ID23-1, Grenoble (*λ*=0.97 Å, *T*=100 K, ID23). The crystals of *apo* ECF–FolT2 belong to space group P1 (unit cell parameters: *a*=88.82 Å, *b*=95.32 Å, *c*=107.57 Å, *α*= 83.45°, *β*=65.75° and *γ*=61.99°). The crystals of ECF–FolT2 grown in the presence of 10 mM MgCl_2_, 10 mM AMP–PNP and 1 μM folate belong to space group P1 (unit cell parameters: *a*=90.07 Å, *b*=97.26 Å, *c*=105.45 Å, *α*= 84.67°, *β*=64.78° and *γ*=62.59°). Crystals of FolT1 were obtained using the hanging-drop vapour-diffusion technique using a reservoir solution of 100 mM Tris, pH 7.5, 24% (v/v) PEG350 MME. These crystals had a rectangular shape and diffracted up to 2.8 Å resolution. Diffraction data were collected at the Swiss Light Source (SLS), Villigen (*λ*=0.92 Å, *T*=100 K, X06SA). The FolT1 crystals belong to space group C 1 2 1 (unit cell parameters: *a*=108.91 Å, *b*=77.54 Å, *c*=89.45 Å, *α*= 90.00°, *β*=116.36° and *γ*=90.00°). All data sets suffered from severe anisotropy and were treated with the diffraction anisotropy server^33^. Data were processed with XDS and molecular replacement was carried out with Phaser MR^34^. For the *apo* structure, molecular replacement with the EcfA–EcfA′ heterodimer structure of *Thermotoga maritima* (PDB ID: 4HLU) yielded a solution for the EcfA and EcfA′ subunit, in which the structures of EcfT and FolT2 were built in by hand with Coot^35^, guided by the structure of ECF–FolT of *L. brevis* (PDB ID: 4HUQ), and with the help of Phenix autobuild^36^, using the AMP–PNP bound structure. To override bias problems Rosetta-based MR^37^ was also used and refinement was performed with Phenix refinement^38^. The Ramachandran statistics are 91.9%, 90.3% and 96.1% for favoured regions for *apo* ECF FolT2, AMP–PNP-bound ECF FolT2 and folate-bound FolT, respectively, and 7.4%, 8.8% and 3.9% for allowed regions. The statistics for data collection and refinement are summarized in Table 1. A stereo view of 2Fo–Fc electron density is shown in Supplementary Fig. 9.

### Substrate-binding assay by intrinsic fluorescence titration

The affinity of FolT1, FolT2 and their mutants for folate was determined by performing an intrinsic fluorescence titration assay at 25 °C (refs 39, 40), using a Spec Fluorlog 322 fluorescence spectrophotomer. The protein was diluted in buffer K to a final concentration of 50 nM (final volume of 800 μl) and added to a 1,000-μl quartz cuvette. After incubation for 5 min, folate was added in 1 μl steps using a Harvard apparatus syringe pump equipped with a 100-μl gastight glass syringe (Hamilton). Using an excitation wavelength of 280 nm, the emission was recorded at 350 nm. After each substrate addition step, 10 s were allowed for mixing and the fluorescence signals were averaged over a time range of 15 s. Data analysis was performed as described previously^39^.

### Reconstitution into proteoliposomes

Purified ECF–FolT2 was reconstituted in proteoliposomes as described previously^41^, using a protein to lipid (w/w) ratio of 1:250 and using liposomes composed of *E. coli* polar lipids and egg phosphatidylcholine (3:1 (w/w) ratio).

### Radiolabelled folate transport assay

To use in the transport assay or the ATPase activity assay, proteoliposomes were thawed and 5 mM of Na_2_-ATP plus 5 mM of MgSO_4_, 5 mM Na_2_-ADP plus 5 mM MgSO_4_, 5 mM Na_2_-ATP plus 5 mM EDTA or 5 mM AMP–PNP plus 5 mM MgSO_4_ were added. To include these compounds in the proteoliposomes, three cycles of flash-freezing in liquid nitrogen and quick thawing of the proteoliposomes were performed. Subsequently, the proteoliposomes were extruded through a 400-nm pore size polycarbonate filter (Avestin, 11 passages). To wash away the external nucleotides, the proteoliposomes were diluted 32 times to a final volume of 8 ml. After centrifugation (45 min, 285,775*g*, 4 °C), the proteoliposomes were resuspended in buffer A to a final concentration of 0.5–1 μg ECF–FolT2 μl^−1^. For each time point in the transport assays, a reaction volume of 200 μl of buffer A supplemented with 5 nM of [3, 5, 7, 9-^3^H] radiolabelled folate and 95 nM of non-radiolabelled folate was incubated at 25 °C while being stirred. Transport was started by adding 1 μg of ECF–FolT2, reconstituted in proteoliposomes. At the indicated time point, 2 ml of stop buffer (ice-cold buffer A) was added and the reaction was rapidly filtered over a BA-85 nitrocellulose filter. After washing the filter with another 2 ml of stop buffer, the filter was dried for 1 h at 80 °C. Subsequently, the filter was dissolved in 5 ml of Filter Count scintillation liquid (PerkinElmer) and the levels of radioactivity were determined using a PerkinElmer Tri-Carb 2800 TR isotope counter. For the efflux experiments, uptake of radiolabelled folate by MgATP-loaded proteoliposomes was allowed for 16 min, after which 5 mM of MgATP, 5 mM MgADP, 5 mM ATP plus 5 mM EDTA or 100 μM non-radiolabelled folate was added and efflux was followed for an additional 8 min.

### ATPase activity assay

The ATPase activity of ECF–FolT2 reconstituted in proteoliposomes (loaded with 0 or 100 nM of folate as described above) was measured by using a coupled enzyme assay, in which the amount of ADP produced (and thus the amount of ATP hydrolysed) is coupled stoichiometric with the oxidation of NADH^42^. The assay was performed at 30 °C in a 96-well plate and the absorbance at 340 nm was measured by a Synergy MX-96-well plate reader (BioTek Instruments, Inc.). A volume of 200 μl of reaction solution per well contained 50 mM KPi, pH 7.5, 200 mM NaCl, 5.2 nM (1.2 μg) of ECF–FolT2, 4 mM sodium phosphoenolpyruvate, 0.3 mM NADH and 3.5 μl of pyruvate kinase/lactic dehydrogenase enzyme mixture from rabbit muscle (Sigma-Aldrich) in 50% glycerol. The reaction solutions were supplemented with 0 or 100 nM folate as indicated (folate was thus present both on the inside and the outside of the proteoliposomes in the 100 nM folate condition). After incubation of the reaction solutions for 3 min at 30 °C, 1 mM of MgATP, pH 7.5, was added to each of the reactions and the absorbance of NADH at 340 nm was followed for 7 min. The ATPase activity was expressed in μmol of ATP hydrolysed per min per mg of ECF–FolT2).

## Additional information

**How to cite this article:** Swier, L. J. Y. M. *et al.* Structural insight in the toppling mechanism of an energy-coupling factor transporter. *Nat. Commun.* 7:11072 doi: 10.1038/ncomms11072 (2016).

## Supplementary Material

Supplementary Information Supplementary Figures 1-9, Supplementary Table 1, Supplementary Note 1 and Supplementary References.

## Figures and Tables

**Table 1:** Data collection, phasing and refinement statistics.

	**FolT1**	**AMP**–**PNP bound ECF**–**FolT2**	***apo*** **ECF**–**FolT2**
*Data collection*			
Space group	C121	P1	P1
Cell dimensions			
*a*, *b*, *c* (Å)	108.91, 77.54, 89.45	90.07, 97.26, 105.45	88.82, 95.32, 107.57
*α*, *β*, *γ* (°)	90.0, 116.36, 90.0	84.67, 64.78, 62.59	83.45, 65.75, 61.99
Resolution (Å)	43.0–3.01 (3.32–3.01)*	43.9–3.30 (3.40–3.30)*	49.0–3.00 (3.07–3.00)*
*R*_merge_	0.12 (0.38)*	0.026 (0.86)*	0.04 (0.92)*
*I*/σ*I*	5.3 (2.18)*	12 (1.10)*	10.5 (1.65)*
Completeness (%)	82.3 (44)*	94.4 (78.8)*	92.6 (86.6)*
Redundancy	2 (1.8)*	1.8 (1.7)*	1.8 (1.72)*
			
*Refinement*			
Resolution (Å)	3.01	3.30	3.00
No. of reflections	10,960	33,723	41,549
*R*_work_/*R*_free_	0.235/0.286	0.252/0.294	0.232/0.279
No. of atoms	2,456	15,554	15,398
Protein	2,392	15,430	15,398
Ligand/ion	64	124	—
Water	—	—	—
*B*-factors			
Protein	36	159	98
Ligand/ion	17	158	—
Water	—	—	—
R.m.s. deviations			
Bond lengths (Å)	0.011	0.006	0.005
Bond angles (°)	1.429	1.404	1.263

For each structure, one crystal was used for data collection.

^*^Values in parentheses are for the highest-resolution shell.
