# Supplementary material for: Structural insight in the toppling mechanism of an energy-coupling factor transporter
Source: Nat Commun. 2016 Mar 30;7:11072. doi: 10.1038/ncomms11072 (PMC4820897; doi:10.1038/ncomms11072)
Supplement: Supplementary Information — Supplementary Figures 1-9, Supplementary Table 1, Supplementary Note 1 and Supplementary References. [file ncomms11072-s1.pdf]

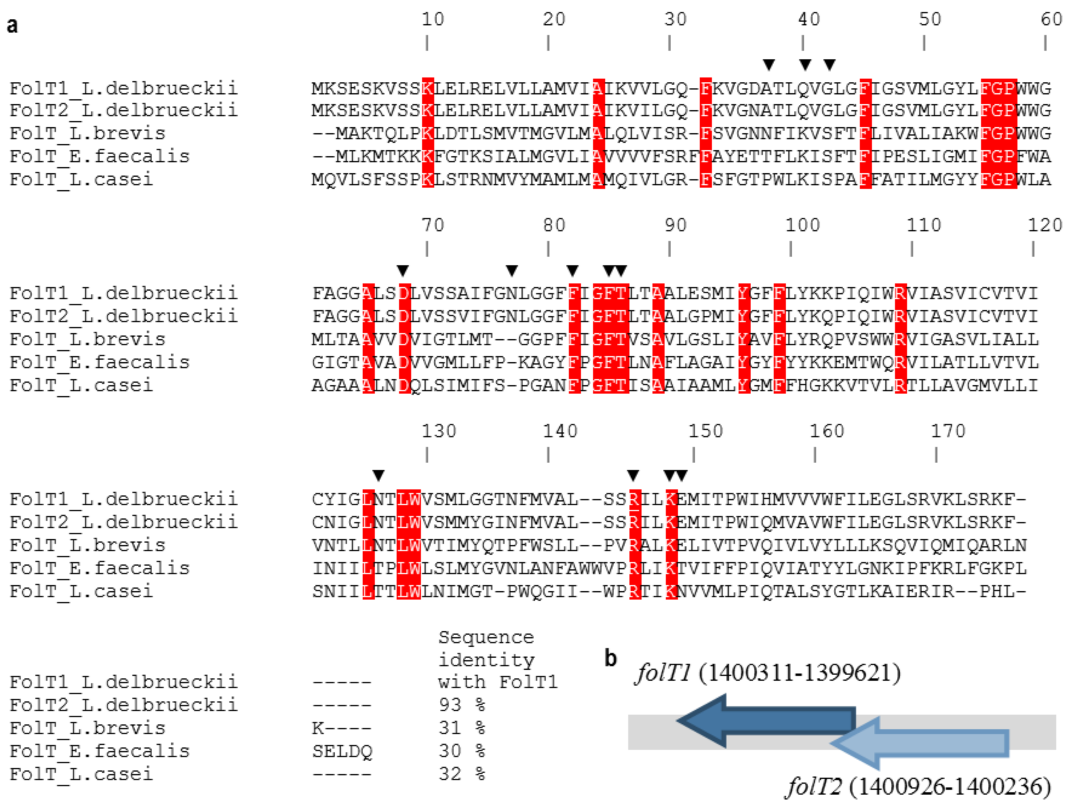

Supplementary Figure 1: Sequence alignment of FolT proteins. (**a**) Sequence alignment of FolT1 from *L. delbrueckii* with FolT2 of *L. delbrueckii* and FolT of *Lactobacillus brevis* (WP_01166775), *Enterococcus faecalis* (WP_002385065) and *Lactobacillus casei* (WP_003567081). Invariant residues are highlighted in red. The FolT1 residues involved in the interaction with folate are indicated by black triangles. At the end of the sequences, the sequence identity of the others protein with FolT1 is given. (**b**) Schematic representation of the location of *folT1* and *folT2* on the genome of *L. delbrueckii*. The genes overlap but make use of different reading frames.

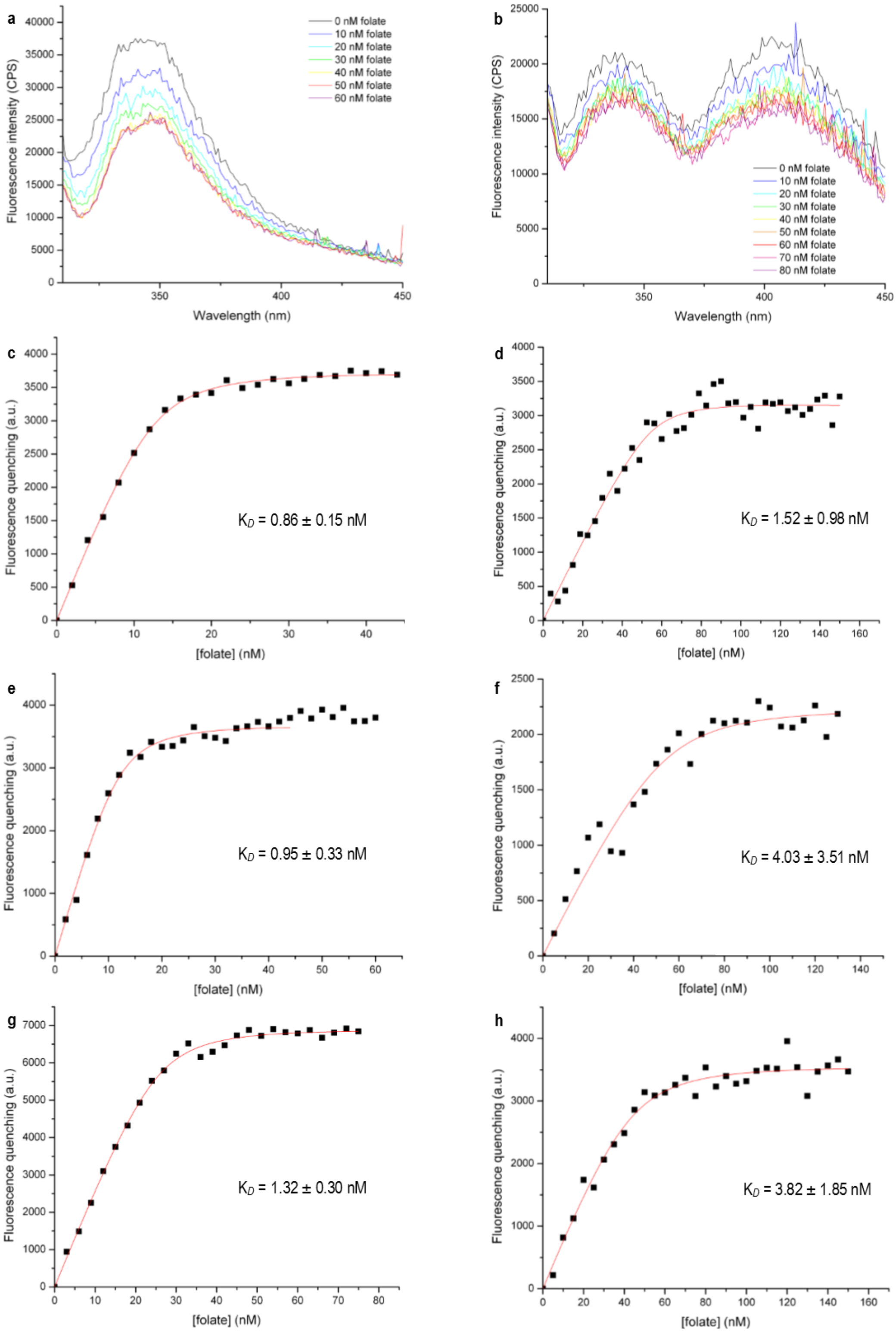

Supplementary Figure 2: Folate binding to FolT1 and FolT2. (**a**) Fluorescence emission spectrum (excitation wavelength of 280 nm) of FolT1 (50 nM) in the absence of folate (black trace) and in the presence of various concentrations of folate up to 60 nM (purple trace). (**b**) Same as in (**a**) with FolT2, with the concentrations of folate ranging from 0 nM (black trace) to 70 nM (grey trace). (**c**, **e**, **g**) Fluorescence titration (excitation wavelength of 280 nm, emission wavelength of 350 nm) showing the quenching of the intrinsic fluorescence of FolT1 (50 nM) as function of the concentration of folate. The data was fitted to a single site binding model and the determined K_D value is indicated together with the error of the fit. (**d**, **f**, **h**) Same as in (**c**, **e**, **g**) with 50 nM of FolT2.

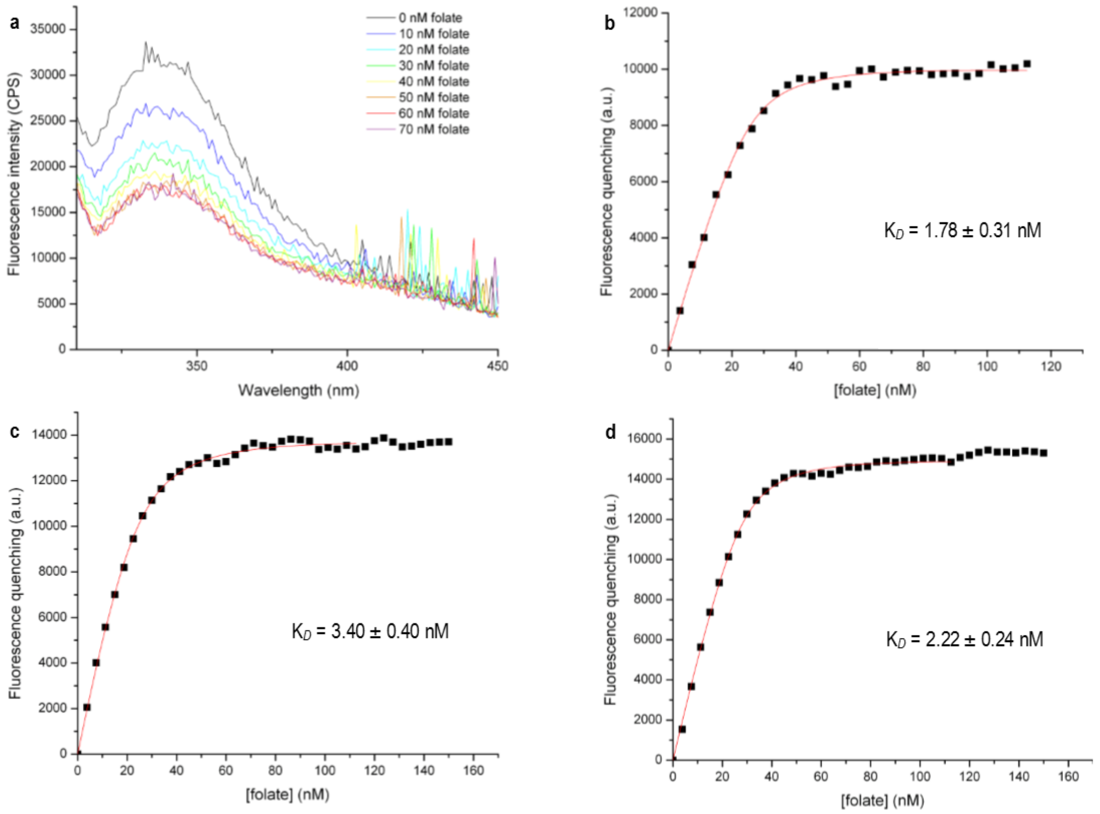

Supplementary Figure 3: Folate binding to FolT1 Y122N and FolT2 N122Y. (**a**) Fluorescence emission spectrum (excitation wavelength of 280 nm) of FolT1Y122N (50 nM) in the absence of folate (black trace) and in the presence of various concentrations of folate up to 70 nM (purple trace). (**b-d**) Fluorescence titration (excitation wavelength of 280 nm, emission wavelength of 350 nm) showing the quenching of the intrinsic fluorescence of FolT1 Y122N (50 nM) as function of the concentration of folate. The data was fitted to a single site binding model and the determined K_D value is indicated together with the error of the fit.

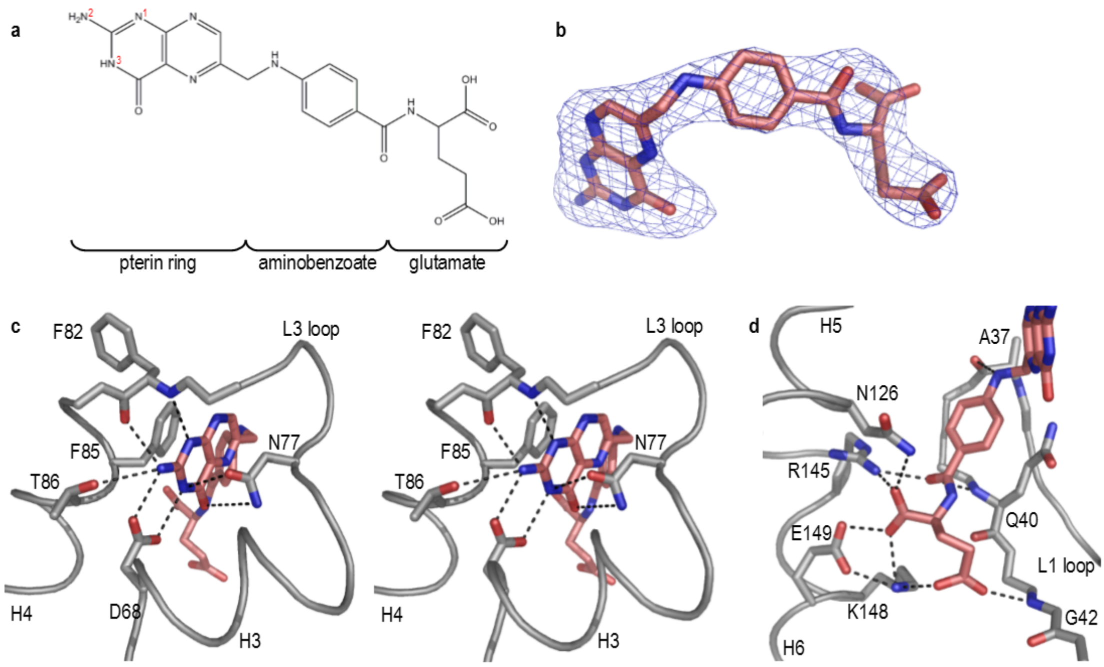

Supplementary Figure 4: Crystal structure of folate-bound FolT1. (**a**) Chemical structure of folate. (**b**) 2Fo-Fc electron density at 2σ in blue contouring folate as bound in the substrate-binding pocket. (**c**) Stereo view of the residues involved in binding the pterin ring of folate. The backbone is shown in ribbon representation and the hydrogen bonds are depicted by black dashed lines. (**d**) FolT1 residues involved in binding of the aminobenzoate and glutamate moieties of folate.

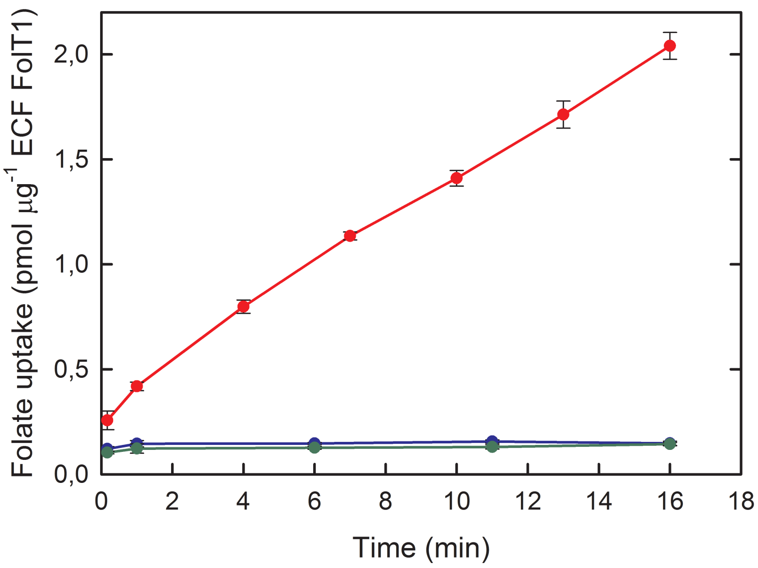

Supplementary Figure 5: Transport of radiolabeled folate by ECF-FolT1 in proteoliposomes. Uptake activity of ECF-FolT1 in proteoliposomes loaded with 5 mM of MgATP (red), 5 mM MgADP (blue) or 5 mM Na_2ATP plus 5 mM EDTA (green). The error bars show the standard deviations from three independent measurements.

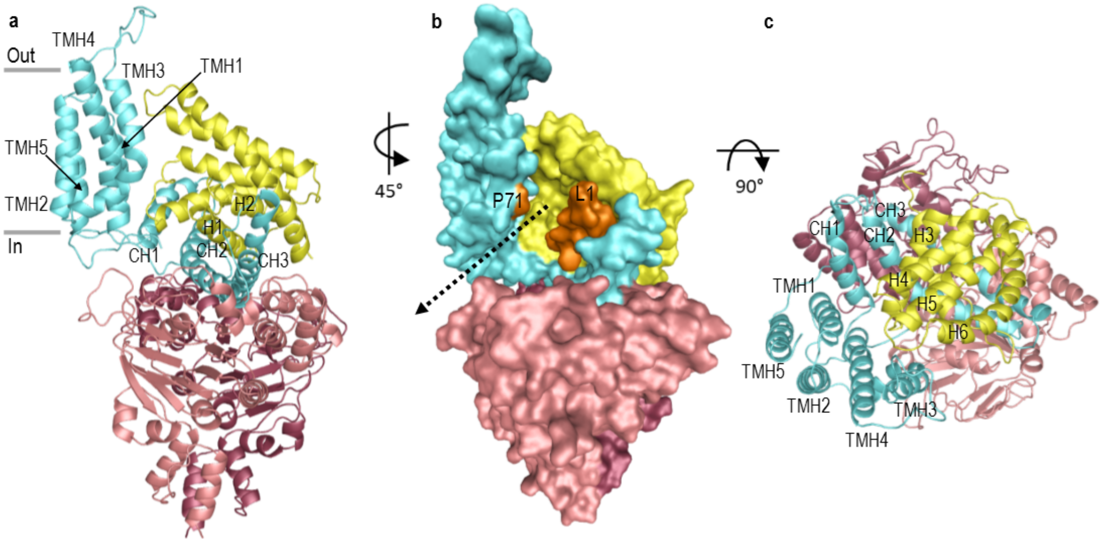

Supplementary Figure 6: Crystal structure of *apo* ECF- FolT2. This figure is the equivalent of Fig. 3 of the main text for the AMP-PNP-bound structure. (**a**) Cartoon representation of ECF-FolT2 from the plane of the membrane, with EcfA and EcfA' colored in two shades of red, EcfT in cyan and FolT2 in yellow. The transmembrane helices of EcfT are indicated by TMH1-5 and the three coupling by CH1-3. (**b**) Surface representation using the subunit colors from panel (**a**). Loop L1 of FolT2 and Proline 71 (P71) in TMH3 of EcfT are colored orange. The pathway leading from the open folate-binding cavity to the cytosol is indicated by the dashed arrow. (**c**) View along an axis perpendicular to the membrane.

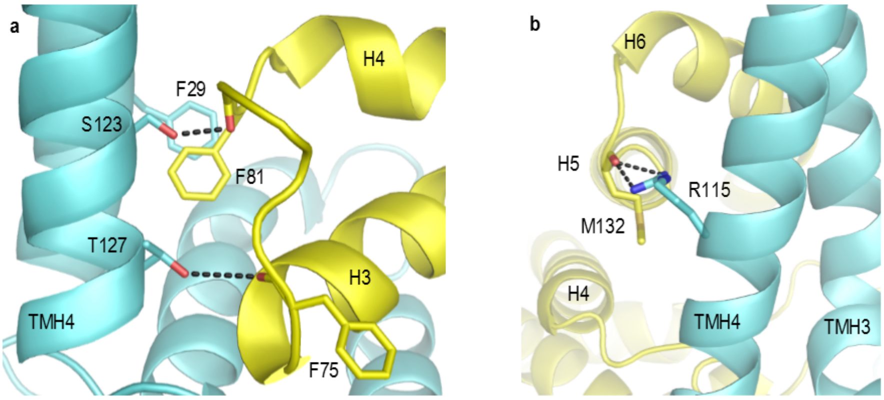

Supplementary Figure 7: Interactions between the membrane domain of EcfT and FolT2 in the AMP-PNP-bound structure. (**a**) Interactions between the serine- and threonine-rich stretch of EcfT and loop L3 of FolT2. (**b**) Interaction of R115 in EcfT with the C-terminus of H5 in FolT2.

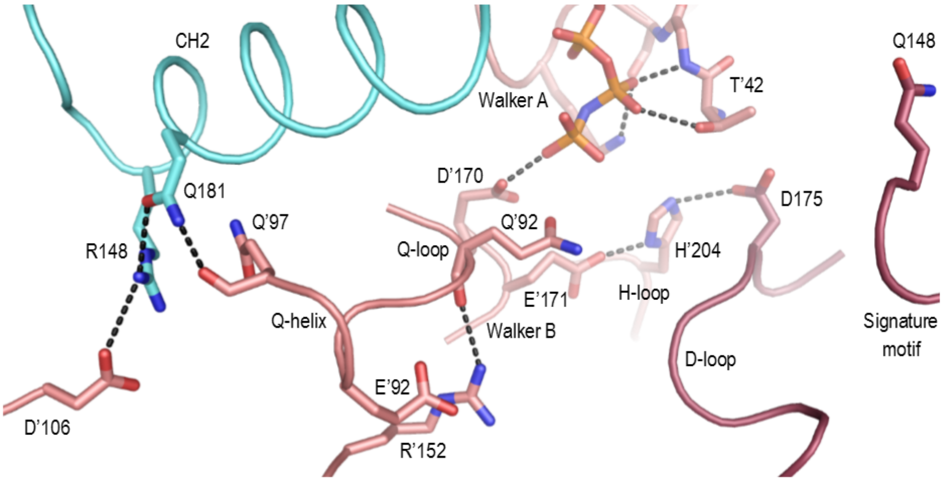

Supplementary Figure 8: Interaction network in EcfA'. The conserved R184 at the C-terminus of coupling helix CH2 interacts via the Q-helix with the AMP-PNP binding site on EcfA'.

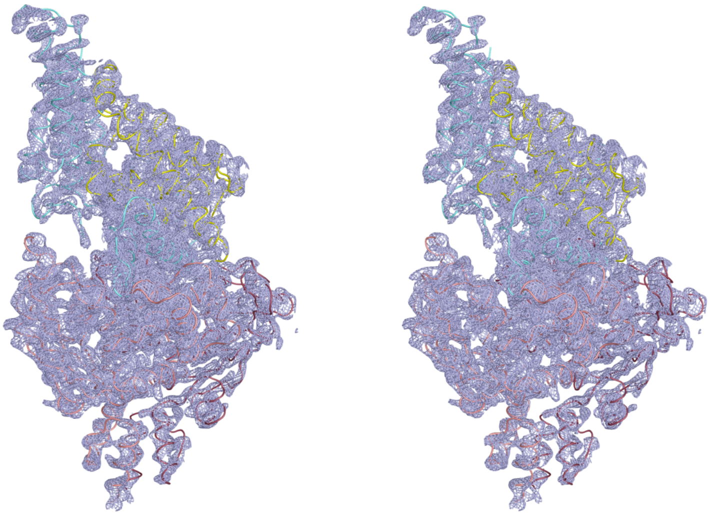

Supplementary Figure 9: Stereo view of 2fo-fc electron density of the ECF-FolT2 complex with AMP-PNP bound, countered at 1.5 sigma

Supplementary Table 1: Primers to introduce mutations in FolT1 and FolT2

Mutation	Primer sequence	
FolT1 Y122N	Forward primer	5’-CACGGTAATCTGCAATATCGGCTTGAACAC
	Reverse primer	5’-GTGTTCAAGCCGATATTGCAGATTACCGTG
FolT2 N122Y	Forward primer	5’-CACGGTAATCTGCTACATCGGCTTGAACAC
	Reverse primer	5’-GTGTTCAAGCCGATGTAGCAGATTACCGTG

Supplementary Note

Interactions between folate and FolT1 (related to Fig. 1 of the main text and Supplementary Fig. 3)

The N^1 atom from the pterin ring forms hydrogen bonds with the backbone amine group of the conserved F82 (in some species replaced with Y) in the L3 loop, while the N^2H_2 group can form hydrogen bonds with the backbone O atom of F82, and the side chains of the conserved residues D68 and T86 in H3 and H4, respectively. The side chain of D68 also forms a hydrogen bond with the N^3H group. The side chain of N77 from the L3 loop forms hydrogen bonds with the N^3H and the O atom of the pterin ring, while being coordinated via backbone interactions by G80 in the same loop. Simultaneously, the pterin ring has π-π interactions with the side chain of the conserved F85 (in some species replaced with Y) in H4. The backbone O atom of A37 in the L1 loop forms a hydrogen bond with the amine group of the aminobenzoate moiety, while the benzoate is stacked by Q40 (often replaced with K or R in other species) from the L1 loop. Q40 is coordinated in this position by a hydrogen bond formed between its sidechain and the backbone O atom of D36. At the same time, the backbone amine of Q40 and the side chain of conserved Arg145 in H6 form hydrogen bonds with the carbonyl O atom of the benzoate moiety. The backbone carboxyl group of the glutamate moiety can form four hydrogen bonds with the side chains of N126 in H5, and R145, conserved K148 (in some species replaced with Q) and E149 (in some species replaced with Q or N) in H6; additionally the side chain carboxyl group forms two hydrogen bonds with the side chain of K148 and the backbone O atom of G42 in the L1 loop. Furthermore, there are interactions between the side chains of K148 and E149 and the backbone of R145 and K148. All of the coordinating residues located in the helices interact with folate via their side chains, whereas the residues located in the L1 and L3 loops interact via their backbones.

Although the interactions between the invariant residues D68, F85, T86, R145 and K148 and folate were also found in the recent structure of FolT from *E. faecalis*[1] (30 % identity with FolT1, **Supplementary Fig. 1a**), there are also conspicuous differences. Four residues involved in folate binding in the enterococcal protein (R26, K36, N117 and T121) are not conserved in FolT1, where the corresponding residues Q31, Q40, Y122 and N126 are either not involved in folate binding (Q31 and Y122), or interact with folate in a different way (Q40 and N126). Conversely, N77 interacts tightly with the pterin ring in FolT1, but this residue is not conserved in the enterococcal protein. These differences highlight that caution must be taken when inferring mechanistic details from comparisons of structures from different proteins with low sequence identity and originating from different organisms. In this light it is noteworthy that FolT1 and FolT2 from *L. delbrueckii* differ only in twelve residues, none of which affect the interactions with folate or the association with EcfT (see below), with the only possible exception of position 122, which is a tyrosine in FolT1 and an asparagine in FolT2. The latter could provide an additional hydrogen bond with the carboxyl side chain group of the glutamate moiety of folate.

Interactions between the ATPases and AMP-PNP (related to Fig. 6 of the main text and Supplementary Fig. 7)

An aromatic residue in the A-loop (F13 in EcfA, Y'12 in EcfA') forms a π-π interaction with the adenosine ring. K46/K'46, the backbones of two glycines and N42/T'42 in the Walker A motif interact with the phosphate groups. E169/E'171 of the Walker B motif interacts with the conserved H203/H'204 in the H-loop, which in turn interacts with the D175 of the D-loop in EcfA' site. The Q-loop is followed by the Q-helix formed by Q96/Q'97 and D94/E'95, which is a conserved feature in ECF transporters[2]. This Q-helix has been proposed to form the link between the ATP hydrolysis site and the coupling helices of EcfT[2]. In our AMP-PNP-bound structure, the conserved residues R184 and R225 at the C-terminal ends of coupling helices 2 and 3, are inserted into the EcfA' and EcfA subunits, respectively, where they

interact with D106' and D105, respectively (**Fig. 6a,d**). The two arginines were first predicted to be important for coupling based on mutagenesis studies[3]. In EcfA', R184 also interacts via Q'181 with Q'97 of the Q-helix, while this network is not observed in EcfA. In the ECF-FolT and ECF-HmpT structures of *L. brevis*, the same arrangement was shown[4,5]. However, in our structure we also observe how E'95 on the opposite side of the Q-helix interacts via R'152 with the backbone of Q'92 (**Fig. 6d**). This provides a connection from the ATP binding site to the coupling helices, making it possible to move the coupling helices according to events taking place in the ATP binding site.

Supplementary references

1. Zhao, Q. *et al.* Structures of FolT in substrate-bound and substrate-released conformations reveal a gating mechanism for ECF transporters. *Nat. Commun.* **6,** 7661 (2015).
2. Karpowich, N. K. & Wang, D.-N. Assembly and mechanism of a group II ECF transporter. *Proc. Natl. Acad. Sci. U. S. A.* **110,** 2534–9 (2013).
3. Neubauer, O. *et al.* Two essential arginine residues in the T components of energy-coupling factor transporters. *J. Bacteriol.* **191,** 6482–8 (2009).
4. Xu, K. *et al.* Crystal structure of a folate energy-coupling factor transporter from Lactobacillus brevis. *Nature* **497,** 268–271 (2013).
5. Wang, T. *et al.* Structure of a bacterial energy-coupling factor transporter. *Nature* **497,** 272–276 (2013).
